# Trichoptera of Canada

**DOI:** 10.3897/zookeys.819.31140

**Published:** 2019-01-24

**Authors:** Cory S. Sheffield, Jeremy R. deWaard, John C. Morse, Andrew K. Rasmussen

**Affiliations:** 1 Royal Saskatchewan Museum, 2340 Albert Street, Regina, Saskatchewan, S4P 2V7, Canada Royal Saskatchewan Museum Regina Canada; 2 Centre for Biodiversity Genomics, University of Guelph, Guelph, Ontario, N1G 2W1, Canada University of Guelph Guelph Canada; 3 Department of Plant & Environmental Sciences, Clemson University, E-143 Poole Agricultural Center, Clemson, South Carolina 29634-0310, USA Clemson University Clemson United States of America; 4 Center for Water Resources, College of Agriculture and Food Sciences, Florida A&M University, 113 South Perry-Paige Bldg., Tallahassee, Florida 32307-4100, USA Florida A&M University Tallahassee United States of America

**Keywords:** biodiversity assessment, Biota of Canada, caddisflies, Trichoptera

## Abstract

Trichoptera, or caddisflies, are common members of freshwater ecosystems as larvae and are important indicators of aquatic system health. As such, the species are relatively well studied, with keys available for larvae and adults of many of the taxa occurring in Canada. The number of species recorded from Canada since 1979 (Wiggins 1979) has increased from 546 to 636, an increase of 16.4%. Of those species newly recorded, 17 represent newly described taxa since 1979. Taking into consideration the species likely to be subsequently found in Canada based on records in adjacent parts of the United States and results from DNA barcoding, an estimated 129–181 species remain to be documented in Canada.

Trichoptera, or caddisflies, is a species-rich group of holometabolous insects with more than 16,000 extant species worldwide (Morse 2018 and see http://entweb.sites.clemson.edu/database/trichopt/), the seventh largest order of insects (Adler and Foottit 2017). The order originated approximately 234 Mya (Malm et al. 2013), and is considered the sister group to Lepidoptera (butterflies and moths) (Morse 1997, Wiggins 1998, Misof et al. 2014). Trichoptera have larvae and pupae that are almost exclusively aquatic (Morse 2017); *Philocascademita* Ross (Limnephilidae) and Manophylax (Madeophylax) spp. are some exceptions (Schmid 1998, Chuluunbat et al. 2010). Mackay and Wiggins (1979) suggest that the high trichopteran diversity was driven by the larval secretion of silk, which provided opportunities to exploit different ecological niches. Wiggins (1998) further speculated that the diversification of Trichoptera has taken place entirely within aquatic habitats, due to larvae being able to exploit food resources in new ways thanks to their diverse case/retreat/filter-net construction. The legs of pupal Trichoptera are modified for the water to land transition, allowing pharate adults to swim to the water surface and sometimes to land, with specialized claws used for crawling on stones or plants (Friedrich and Kubiak 2018).

Caddisfly larvae are well known for their underwater architecture, with some taxa constructing elaborate cases out of a range of materials. The behaviours, type of materials used, and the shape of the case often being diagnostic for trichopteran identification (Weaver and Morse 1986, Wiggins 1996, 2004). Some workers (Wiggins and Wichard 1989, Frania and Wiggins 1997, Wiggins 1998, Kjer et al. 2001, Holzenthal et al. 2007, Malm et al. 2013) have recognized three suborders of Trichoptera corresponding to larval construction behaviours (and see Morse 1997, 2017). Annulipalpia (or “fixed-retreat-makers”) attach themselves to substrates using silk which often also acts to gather food items from the passing water currents. Integripalpia contain species with “portable-case-making” larvae, constructing tubular cases of various materials (e.g., leaves, wood, small pebbles) held together with silk. “Spicipalpia”, consist of larvae that construct closed, semi permeable cocoons for pupation, but which exhibit a wide range of larval behaviours, including some free-living predatory larvae and herbivorous forms that build portable enclosures to provide shelter while they graze, but do not build traditional tube cases or filtering nets like the majority of caddisfly taxa (Malm et al. 2013). Morse (1997, 2017) provided more specific details on the taxa historically included within “Spicipalpia” (i.e., Rhyacophiloidea, Glossosomatoidea, Hydroptiloidea), but the most recent phylogenies (e.g., Kjer et al. 2016, Morse et al. in preparation) consider these families to be basal lineages of Integripalpia. For this faunistic summary, we structure Table 1 to reflect the recent summary of trichopteran higher classification provided by Holzenthal et al. (2011), modified for taxa found in Canada.

**Table 1.** Census of Trichoptera in Canada^1^.

Taxon^1^	No. species reported by Wiggins (1979)	No. species currently known from Canada^2^	No. BINs^3^ available for Canadian species	Est. no. undescribed or unrecorded species in Canada^4^	General distribution by ecozone^5^	Information sources^6^
**Suborder Annulipalpia**						
**Superfamily Philopotamoidea**						
Philopotamidae	13	14	20	1–13	most ecozones	Schmid 1982, Armitage 1991
**Superfamily Psychomyioidea**						
Dipseudopsidae ^7^	0	3	4	1–2	Atlantic Maritime, Mixedwood Plains, Prairies	Schmid 1983, Schuster and Hamilton 1984, Sturkie and Morse 1998
Polycentropodidae	36	37	32	6	most ecozones	Nimmo 1986, Armitage and Hamilton 1990, Neboiss 1993, Chamorro and Holzenthal 2010
Psychomyiidae	2	4	2	1–4	Atlantic Maritime, Mixedwood Plains, Prairies	Schmid 1983, Armitage and Hamilton 1990
**Superfamily Hydropsychoidea**						
Hydropsychidae ^8^	48	56	62	10–15	most ecozones	Schmid 1968, Gordon 1974, Nimmo 1987, Geraci et al. 2010, Burington 2011
**Suborder Integripalpia – “Spicipalpia**”						
**Superfamily Glossosomatoidea**						
Glossosomatidae	21	26	22	8–15	most ecozones	Ross 1956, Nimmo 1974, 1977, Schmid 1982, Wymer and Morse 2000, Etnier et al. 2010, Robertson and Holzenthal 2013, Genco and Morse 2017
**Superfamily Hydroptiloidea**						
Hydroptilidae	52	75	78	10	most ecozones	Kingsolver and Ross 1961, Denning and Blickle 1972, Ito et al. 2014, Harris and Flint 2016
Ptilocolepidae ^9^	?	2	1	0	Pacific Maritime, Newfoundland Boreal; possibly Mixedwood Plains, Boreal Shield, Atlantic Maritime	Ito et al. 2014
**Superfamily Rhyacophioidea**						
Rhyacophilidae	57	65	36	15	most ecozones	Schmid 1966, 1970, 1981, Nimmo 1971, Prather and Morse 2001
**Suborder Integripalpia – Brevitentoria**						
**Superfamily Leptoceroidea**						
Calamoceratidae	2	2	1	0	Pacific Maritime, Western Interior Basin, Montane Cordillera, Mixedwood Plains	Bowles and Flint 1997, Schmid 1998
Molannidae ^10^	7	6	3	0	most ecozones, except Atlantic Maritime and Arctic	Roy and Harper 1980, Schmid 1983, 1998
Leptoceridae	56	68	118	30–50	most ecozones	Yamamoto and Wiggins 1964, Holzenthal 1982, Floyd 1995, Glover 1996, Manuel 2010
Odontoceridae	4	5	3	0	Mixedwood Plains, Atlantic Maritime	Schmid 1983, Parker and Wiggins 1987
**Superfamily Sericostomatoidea**						
Beraeidae	1	1	0	1	Mixedwood Plains	Wiggins 1954, Schmid 1998
Helicopsychidae	1	1	5	3	most ecozones	Moulton and Stewart 1996, Johanson 2002
Sericostomatidae	2	2	2	2	Mixedwood Plains	Ross and Wallace 1974, Schmid 1998, Keth and Harris 2008
**Suborder Integripalpia – Plenitentoria**						
**Superfamily Limnephiloidea**						
Apataniidae	?	16	8	0	most ecozones, except Atlantic Maritime	Schmid 1953, Chen 1992, Flint 2007
Goeridae	?	5	4	2	Atlantic Maritime, Mixedwood Plains, Montane Cordillera, Western Interior Basin, Pacific Maritime	Schmid 1983, 1998
Limnephilidae	179	157	128	30	most ecozones	Parker and Wiggins 1985, Ruiter 1995
Rossianidae	?	1	0	0	Montane Cordillera, Western Interior Basin, Pacific Maritime	Schmid 1998
Thremmatidae	?	16	9	3	Newfoundland Boreal and south of boreal on mainland	Vineyard et al. 2005, Hoemsen et al. 2015
Uenoidae	?	2	1	0	widespread in Canada	Wiggins et al. 1985
**Superfamily Phryganeoidea**						
Brachycentridae	15	16	16	2	most ecozones	Wiggins 1965, Schmid 1983, Flint 1984
Lepidostomatidae	26	30	27	2–3	most ecozones	Weaver 1984, 1988
Phryganeidae	24	26	28	2–5	most ecozones	Wiggins 1956, 1960, 1998
**Total**	**546**	**637**	**611**	**129–181**		

^1^Modified from Holzenthal et al. (2011) and Ito et al. (2014). ^2^Data extracted from Rasmussen and Morse (2018)^3^Barcode Index Numbers (BINs), as defined by Ratnasingham and Hebert (2013). ^4^Estimates based on data in Schmid (1998) and Rasmussen and Morse (2018) and from BINs. ^5^See figure 1 in Langor (2019) for a map of ecozones. ^6^The references cited do not necessarily represent a comprehensive list for each family but rather some of the most significant contributions. See Schmid (1998) for major taxonomic works for Canada to that time and, more importantly, Rasmussen and Morse (2018) provide the most complete species level account of relevant literature with distributional data. BOLD refers to DNA barcode data from the Barcode of Life Data System (www.boldsystems.org). ^7^Dipseudopsidae = Hyalopsychidae of Schmid (1998). ^8^Includes Arctopsychidae of Nimmo (1987) and Schmid (1998). ^9^Records of this family outside of the Pacific Maritime and Newfoundland Boreal are based on single specimen accounts from the literature with no collection information (Ito et al. 2014). ^10^The decrease of one species since 1979 is due to the fact that *Molannacinerea* Hagen, 1861 is now considered nomen dubium.

Trichoptera continue to be the subjects of much taxonomic work largely because this insect order is among the most important and diverse of all aquatic taxa (Holzenthal et al. 2007), exceeded in number in freshwater habitats only by Coleoptera (16,000+ species) and Diptera (51,000+ species) (Morse 2017), and are key elements of freshwater ecosystems for biological assessment and water quality monitoring. Especially because of their importance in freshwater biomonitoring, Trichoptera is one of the few insect orders in which keys exist for both the larvae (e.g., Wiggins 1996, 1998, Morse and Holzenthal 2008) and adults (e.g., Cooper and Morse 1998, Schmid 1998, Wiggins and Currie 2008), though Wiggins (1979) and Morse (2017) indicated that there is a major deficiency in our ability to identify the immature stages.

Trichoptera taxonomy has a rich history in Canada, with workers such Glenn B. Wiggins, Andrew P. Nimmo, and Fernand Schmid laying a solid foundation for ongoing and future work. Many other North America workers continue to contribute to knowledge of the Canadian trichopteran fauna (see Table 1). Since Wiggins’ (1979) summary of the Canadian fauna, Morse (1993) published a checklist of 1653 North American species, which included the fauna of Mexico and Greenland, but did not partition these by country. Schmid (1998) published keys to the genera occurring in Canada and the adjacent United States, which included estimates of numbers of species in each. Unfortunately, precise numbers for species richness in Canada were not provided, though he did provide estimates for Canada and adjacent areas. Most of the data supporting the current assessment (Table 1) are based on an online list of Nearctic Trichoptera (Rasmussen and Morse 2018). Since the time of Wiggins’ (1979) summary, the number of Trichoptera species recorded from Canada has increased from 546 to 636, representing an increase of 16.4%. Of the newly recorded species, 17 were described since 1979.

In his overview of Canadian caddisflies, Wiggins (1979) recognized 18 families of Trichoptera within three superfamilies: Rhyacophiloidea (four families), Hydropsychoidea (three families), and Limnephiloidea (11 families), these corresponding to the suborders ‘Spicipalpia’, Annulipalpia, and Intergripalpia, respectively, and tallied 546 species (Table 1). Since that time, different higher level classification schemes based on phylogenetic analyses have been applied to Trichoptera, both in North America (e.g., Wiggins 1996, Schmid 1998) and globally (Holzenthal et al. 2007, 2011).

The Canadian fauna includes ten superfamilies and 25 families (Table 1; after Holzenthal et al. 2011). Rhyacophiloidea (= Spicipalpia of other authors), as recognized by Wiggins (1979), is now partitioned into three superfamilies: Glossosomatoidea (Glossosomatidae); Hydroptiloidea (Hydroptilidae, Ptilocolepidae [= Hydroptilidae, subfamily Ptilocolepinae of Schmid (1998) and likely Wiggins 1979, so not included in the 1979 work]); and Rhyacophiloidea (Rhyacophilidae). By contrast, Hydropsychoidea is currently applied in a much narrower sense than by Wiggins (1979); it is now represented by a single family, Hydropsychidae, which includes Arctopsychidae of Schmid (1998). Of the other three families included in Hydropsychoidea in Wiggins (1979), Philopotamidae has been placed in Philopotamoidea and Psychomyiidae and Polycentropodidae are now in Psychomyioidea. One additional family within Psychomyioidea is newly recorded in Canada since 1979, Dipseudopsidae [treated as Hyalopsychidae by Schmid (1998)]. An additional five families have been newly recorded from Canada (Wiggins 1996, Schmid 1998, Rasmussen and Morse 2018) based on changes in classification, all within superfamily Limnephiloidea: Apataniidae, Goeridae, Thremmatidae, Uenoidae, and Rossianidae. The latter three families were considered part of Limnephilidae by Wiggins (1979), and Rossianidae was previously recognized as the subfamily Dicosmoecinae of Limnephilidae by Schmid (1998), and likely also by Wiggins (1979). Of the eleven families placed in the Limnephiloidea by Wiggins (1989), ten now reside in different superfamilies: Brachycentridae, Lepidostomatidae, and Phryganeidae have been placed in Phryganeoidea; Calamoceratidae, Molannidae, Leptoceridae, and Odontoceridae placed in Leptoceroidea; and Beraeidae, Helicopsychidae, and Sericostomatidae placed in Sericostomatoidea. These additions and reclassifications account for ca. 40 of the additional species within Canada (Table 1).

DNA barcoding (sensu Hebert et al. 2003) has been applied extensively to the Trichoptera fauna of Canada, especially in northern areas (Zhou et al. 2009, Ruiter et al. 2013) and elsewhere, with a comprehensive global library containing more than 16,000 unique haplotypes already well established (Zhou et al. 2016). The 610 Barcode Index Numbers (BINs; Ratnasingham and Hebert 2013) assigned to the Canadian Trichoptera in the Barcode of Life Data System (BOLD; Ratnasingham and Hebert 2007), seemingly represent 96% of the number of described species known from Canada (Table 1). However, many BINs are not yet associated with described species and, in several cases, the ratio of species to BINs is low. For instance, the families Hydropsychidae, Philopotamidae, Hydroptilidae, Phryganeidae, Helicopsychidae, and especially Leptoceridae all have more BINs than known Canadian species suggesting that there are many additional species in Canada remaining to be documented and highlighting that there is still much opportunity for research on Trichoptera in Canada. However, this may also mean that there is enough variation in the barcode region of COI of some Canadian Trichoptera that multiple BINs exist for an individual species, as has been demonstrated in other insect groups (Gibbs 2018). As stressed by Zhou et al. (2016), BINs are not synonymous with species (although there is typically high congruence) and should not be treated as such.

Estimates of the number of undocumented (undescribed or unreported) species in Canada were made by first considering species that are known from adjacent parts of the USA but not yet recorded from Canada. Such species are likely to occur there based on habitat and climate. Furthermore, we took into consideration the number of BINs reported for each family and the likelihood that some of these represent undocumented species. We conservatively estimate that 129–181 additional species will eventually be found in Canada, meaning that the total Canadian fauna could be >800 species (Table 1). The families with the highest numbers of undocumented species are expected to be Leptoceridae (30–50 species) and Limnephilidae (30).

Almost all Canadian jurisdictions (except Prince Edward Island and Labrador) have checklists or at least some faunistics work. Examples include: Yukon (Nimmo and Wickstrom 1984, Wiggins and Parker 1997), Northwest Territories/Nunavut (Nimmo 1984, Winchester 1984, Cordero et al. 2017), British Columbia (Nimmo and Scudder 1978, 1983, Cannings and Roberts 2007, Cannings 2007, Erasmus et al. 2018), Alberta (Nimmo 2001, Hinchliffe 2010), Saskatchewan (Smith 1975, 1984, Hoemsen et al. 2015), Manitoba (Zhou et al 2009, Ruiter et al. 2013), Quebec (Nimmo 1966, Roy and Harper 1979, 1981), New Brunswick and Nova Scotia (Banks 1930, Peterson and van Eeckhaute 1990, 1992), and the island of Newfoundland (Banks 1908, Marshall and Larson 1982). In addition, an up-to-date online global species list (Morse 2018) is available as is an online Endnote-based literature database with more than 12,000 records (Holzenthal et al. 2012), both highly valuable for study of Trichoptera in Canada. Despite the very good taxonomic foundation and state of knowledge concerning faunal composition, there is still plentiful effort needed in Canada before the fauna is fully known. In particular, there are still major challenges to identify the immature stages of Trichoptera (Wiggins 1979), and DNA barcoding offers a means of associating identifiable adults (male and female) to unidentifiable immature stages (Zhou et al. 2007). Barcode data will also help with understanding phylogenetic relationships (Frandsen et al 2016). There are many areas of Canada that need additional caddisfly sampling, particularly northern areas and remote areas in the south. Increased sampling in areas close to the southern border with the USA is also likely to add new Canadian records. With a comprehensive DNA barcode library for Trichoptera well underway (Zhou et al. 2016), the future for Trichoptera studies globally, and within Canada, looks promising.
